# Biofilm and Pathogenesis-Related Proteins in the Foodborne *P. fluorescens* ITEM 17298 With Distinctive Phenotypes During Cold Storage

**DOI:** 10.3389/fmicb.2020.00991

**Published:** 2020-05-28

**Authors:** Laura Quintieri, Francesca Fanelli, Daniela Zühlke, Leonardo Caputo, Antonio Francesco Logrieco, Dirk Albrecht, Katharina Riedel

**Affiliations:** ^1^Institute of Sciences of Food Production, Italian National Research Council, Bari, Italy; ^2^Institute of Microbiology, University of Greifswald, Greifswald, Germany

**Keywords:** food spoilers, temperature adaptation, biofilm, pathogenicity, genomics, proteomics

## Abstract

In food chain, *Pseudomonas* spp. cause spoilage by reducing shelf life of fresh products, especially during cold storage, with a high economic burden for industries. However, recent studies have shed new light on health risks occurring when they colonize immunocompromised patient tissues. Likewise to *P. aeruginosa*, they exhibit antibiotic resistance and biofilm formation, responsible for their spread and persistence in the environment. Biofilm formation might be induced by environmental stresses, such as temperature fluctuations causing physiological and metabolic changes exacerbating food spoilage (by protease and pigment synthesis), and the production of adhesion molecules, chemotactic or underestimated virulence factors. In order to provide a new insight into phenotypic biodiversity of *Pseudomonas* spoilers isolated from cold stored cheese, in this work 19 *Pseudomonas* spp. were investigated for biofilm, pigments, exopolysaccharide production and motility at low temperature. Only nine strains showed these phenotypic traits and the blue pigmenting cheese strain *P. fluorescens* ITEM 17298 was the most distinctive. In addition, this strain decreased the survival probability of infected *Galleria mellonella* larvae, showing, for the first time, a pathogenic potential. Genomic and proteomic analyses performed on the ITEM 17298 planktonic cells treated or not with lactoferrin derived antibiofilm peptides allowed to reveal specific biofilm related-pathways as well as proteins involved in pathogenesis. Indeed, several genes were found related to signaling system by cGMP-dependent protein kinases, cellulose, rhamnolipid and alginate synthesis, antibiotic resistance, adhesion and virulence factors. The proteome of the untreated ITEM 17298, growing at low temperature, showed that most of the proteins associated with biofilm regulation, pigmentation motility, antibiotic resistance and pathogenecity were repressed, or decreased their levels in comparison to that of the untreated cultures. Thus, the results of this work shed light on the complex pathways network allowing psychrotrophic pseudomonads to adapt themselves to food-refrigerated conditions and enhance their spoilage. In addition, the discovery of virulence factors and antibiotic resistance determinants raises some questions about the need to deeper investigate these underestimated bacteria in order to increase awareness and provide input to update legislation on their detection limits in foods.

## Introduction

The *Pseudomonas* genus shows a wide species diversity and encompasses species isolated in a number types of ecological niches and environments. They occur in soil, water, and marine environments as well as in foods, clinical instruments and medical products (Palleroni, 2010; Raposo et al., 2017). This broad distribution is due to the high genomic complexity and plasticity of these bacteria (Stover et al., 2000); not surprisingly, to date, its taxonomy is still being revised and updated. Only few species are classified opportunistic human pathogens and phytopathogens (*Pseudomonas aeruginosa, Pseudomonas syringae*, and *Pseudomonas cichorii*; de Bentzmann and Plésiat, 2011; Ichinose et al., 2013). However, an increasing number of *Pseudomonas* strains, without the issue of pathogenicity, have displayed multiple drug resistances, raised from horizontal transfer of genetic elements or inheritable mutations, which pose a severe threat to human health (Guzel et al., 2018; Quintieri et al., 2019a). Moreover, the isolation of non-pathogenic pseudomonads strains from clinical human cases has rapidly increased. To this regard, *P. fluorescens* colonizes different environments (such as airways, urinary tracts, blood, and glial cells) demonstrating that human body temperature might not be a barrier to the development of this bacterium (Chapalain et al., 2008). As previously reported, phenotypic variants of psychrotrophic *P. fluorescens* strains grew at high temperature, caused hemolysis by means of phospholipase C and surfactant secretion (Sperandio et al., 2010), modulated the permeability of a Caco-2/TC7 intestinal epithelial model (Madi et al., 2010b), and induced macrophage necrosis in a eukaryotic cell model (Sperandio et al., 2012). These findings indicate the emergence of different novel strains, originating from the environment reservoir and spreading in clinical settings, to elicit a neutrophilic response resulting in significant damage to host tissues, often worsen by resistance to clinical treatments (Madi et al., 2010a; Scales et al., 2014; Mazurier et al., 2015). Phenotypic changes usually occur under the influence of environmental stimuli and enhance colonization ability as well as persistence. In food chain, adaptive behavior of *Pseudomonas* spp. is mainly correlated to biocides resistance and biofilm forming phenotypes (Sousa-Silva et al., 2018; Alvarez-Ordóñez et al., 2019). In biofilm, bacterial cells become tolerant to environmental changes and stresses, recruit nutrients and acquired a social behavior generally coordinated through cell-to-cell communication, also named QS; this signaling network allows the cells to control the population load modulating many of their functions and, depending on the signal concentration to activate or to repress target genes (Papenfort and Bassler, 2016). Signaling molecules were detected in many contaminated food suggesting a role of QS in regulating phenotypes responsible for food spoilage (Martins et al., 2018); synthesis of proteolytic and lipolytic enzymes, pigments, siderophores and other metabolites responsible for unpleasant off-flavors are, indeed, QS regulated (Rul and Monnet, 2015; Yuan et al., 2018a). However, beside their role in spoilage, widely studied for the resulting economic losses, these traits underlay complex microbial functions, putatively involved in adaptive and resistance responses as well as microbial competitiveness. Among these, indeed, pigments modulate the transition to planktonic to biofilm state (Mavrodi et al., 2013), show antimicrobial effects against other microorganisms (Gram, 1993), protect cells from oxidative stress and can act as signaling molecules and virulence factors (Reverchon et al., 2002; Dietrich et al., 2006; Liu and Nizet, 2009). Recently, it has been reported that, in non-pathogenic pseudomonads, the synthesis of pigments, peptidases, and biofilm formation increased at low temperature, putatively to overcome the hurdles imposed by cold stress (Yuan et al., 2018b; Rossi C. et al., 2018; Quintieri et al., 2019a, b). Some of these biological components also showed a high similarity with those involved in pathogenesis and resistance mechanisms in the opportunistic pathogen *P. aeruginosa* (Quintieri et al., 2019b), although their role in food spoilage pseudomonads is not yet completely clear. In the study of bacterial persistence, the application of proteomic techniques and the development of information-rich databases have been helping to reveal metabolic pathways involved in a specific physiological state and to provide direct information on druggable targets (Sulaiman and Lam, 2019). Thus, comparative proteomic under treatments with novel antibiofilm molecules (Rasamiravaka et al., 2015; Chan et al., 2016; Caputo et al., 2018; Li et al., 2018; Ahmed et al., 2019) is a high-throughput tool to reveal major putative targets that are directly or indirectly involved in the expression phenotype by different microorganisms (Chatrath et al., 2018; Qayyum et al., 2019).

In light of these considerations, 19 *Pseudomonas* spp. strains isolated from cold stored cheeses were herein investigated for their phenotypic diversity at low temperature; the focused traits were biofilm, pigments, exopolysaccharide (EPS) production as well as motility. The *Pseudomonas* spp. strain exhibiting marked differences in phenotypic characteristics was assayed in *Galleria mellonella* model infection test and further investigated by genomic and proteomic analyses in presence or absence of sub-lethal concentrations of BLFPs. The treatment with these peptides provided a suitable antibiofilm strategy against the target strains and allowed to highlight specific molecular mechanisms underlying spoilage, persistence and pathogenic potential of *Pseudomonas* spp.; the revealed protein determinants and related pathways could be further exploited to implement more effective strategies for their control in the environment.

## Experimental Section

### Bacterial Strains and Culture Conditions

*Pseudomonas fluorescens* NCPPB 1964 from clogged tap water filter, and *P. fluorescens* (ITEM 17299, ITEM 17298, PS37, 84094), *P. gessardii* (PZ20, PS36, 2A), *P. fragi* (PS4, PS20, PS25), *P*. *lundensis* 25E and *P. taetrolens* 26A from High Moisture Mozzarella Cheese (Baruzzi et al., 2012; Quintieri et al., 2013, 2015; Caputo et al., 2015), or ricotta cheese (*P. fluorescens* RC1, RC2, RC3, RC4, RC5, and *P. putida* RC10) were maintained at −80°C as pure stock cultures in Nutrient Broth (NB; Oxoid S.p.A., Rodano, Milan, Italy) supplemented with glycerol 30% (vol/vol). The strains were routinely refreshed (30°C, 24 h) by streaking onto Luria Bertani (LB; Sigma Aldrich, Milan, Italy) agar, and cultivated for 16 h at 30°C and 150 strokes into 5 mL of LB broth to prepare the bacterial inocula for the subsequent experiments.

### Phenotypic Analysis

#### Pigment Productions

Cell suspensions of each *Pseudomonas* spp. strain were spotted (2.5 μL; 1 × 10^8^ CFU/mL) in triplicate onto King A and King B agar Petri dishes (Sigma Aldrich) and minimal medium M63 agar (M63: ammonium sulfate, 2 g/L; potassium phosphate monobasic, 13.6 g/L; ferrous sulfate heptahydrate, 0.5 mg/L; glucose, 2 g/L; magnesium sulfate, 1 mM; casamino acids, 5 g/L and agar, 16 g/L) and incubated at 15°C for 1 week; resulting colonies were also examined under Wood’s lamp.

#### Biofilm Formation

M63 medium in sterile 96-well microtiter plates (Corning^®^, NY, United States) was inoculated in triplicate from each overnight LB-grown culture, diluted 1:100 in minimal M63 medium. Not inoculated M63 was used as negative control. The microtiter plates were then incubated at 15°C for 72 h. After removing planktonic cells, biofilm biomass was stained with crystal violet (CV) and solubilized with 30% acetic acid (v/v); then, its absorbance (CV Abs570 nm) was measured at 570 nm (O’Toole, 2011) at 24, 48, and 72 h.

#### Motility Assays

The liquid cultures of each strain, incubated at both experimental temperatures, and displaying biofilm biomass values higher than 2.0 (as CV Abs_570 nm_), were evaluated in triplicate for swarming, swimming and twitching assays at 24, 48, and 72 h of incubation at 15°C; two strains with CV Abs_570 nm_ < 0.5 were also considered.

Swarming and swimming experiments were performed in polystyrene Petri dishes (50 mm diameter) containing 10 mL of M63 with 0.5 and 0.3% of agar, respectively, as previously described (Quintieri et al., 2019b). Twitching motility was evaluated on M63 minimal medium supplemented with 1 mM MgSO_4_, 0.2% glucose (w/v), 0.5% casamino acids (w/v) and 1% agar (w/v) and inoculated at the bottom of the agar-dish interface. The plates were incubated at 15°C and at the selected times biofilm was quantified as described in Quintieri et al. (2019b).

#### Exopolysaccharides (EPS) Production by Congo Red Binding Assay

*Pseudomonas* spp. cultures, selected for motility assays, were spotted (5 μL) onto Petri dishes containing Congo red agar (10 g/L tryptone, 40 μg/mL Congo red (Sigma), 15 μg/mL Coomassie brilliant and 1.5% agar) and incubated at 15°C for 72 h. The interaction of Congo red with EPS produced the appearance of red colonies (biofilm producers). EPS production was also detected in swimming assay performed in M63 supplemented with 40 μg/mL Congo Red and 15 μg/mL Coomassie brilliant, as described above. In order to quantify EPS, selected cells (1 × 10^3^ CFU/mL) were cultivated in M63 broth for 72 h. At the end of incubation each culture was centrifuged at *10397 ×* rpm for 5 min at room temperature, and the supernatant was discarded. The pellets were resuspended in 1 mL of 40 μg/mL Congo Red and incubated with vigorous shaking at 15 min intervals of time for at least 90 min at room temperature. The samples were again centrifuged (9600 × rpm, 5 min) and the OD of the supernatants was measured at 490 nm. A standard curve was constructed by measuring the OD_490 nm_ of Congo Red at 40, 20, 10, 5, and 2.5 μg/mL; un-inoculated medium was used as a blank and as diluent for the standard curve.

### Determination of *Pseudomonas fluorescens* Infection in *Galleria mellonella* Model

Waxmoth (*G. mellonella*) larvae at the 5^th^ or 6^th^ instar stage (*ca.* 2–3 cm in length, *ca.* 200 mg each) were obtained from a local commercial supplier and were used immediately. Two selected food spoilage *P. fluorescens* strains (displaying the highest and lowest scores for the assayed phenotypic traits, above all biofilm formation and pigmentation) and *P. aeruginosa* DSM 939 (positive control) were grown in M63 or LB medium at 15 and 37°C for 24 or 12 h, respectively. The cultures were centrifuged and washed twice with 10 mM phosphate-buffered saline (PBS; pH 6.5); the inoculum was obtained by resuspending cells in PBS (Sigma-Aldrich, Italy) to an optical density at OD_600 nm_ of 0.3, (corresponding to approximately 8 Log CFU/mL). Before injection, the *Galleria* larvae were separated into Petri dishes in groups of 20 and anesthetized by placing on ice for approximately 30 min. Last left prolegs were disinfected by using sterile cotton plugs dipped in ethanol (70%, v/v), and injected with 10 μL of each bacterial suspension through a 100 μL Hamilton syringe with a 30-gauge needle sterilized by autoclave. New sterilized syringes and needles were used for injection of each bacteria suspension. For each bacterial strain injected, groups containing 7 larvae were used. Two negative controls were also used for each experiment: a no-injection control (to control for background larvae mortality) and a PBS buffer control in which larvae were injected with 10 μL of PBS buffer to ensure that death was not due to the injection trauma. Experiments were repeated twice using larvae from two different batches. After injection, Petri dishes were incubated at 30°C and inspected at 12 h post-injection intervals to record morbidity and mortality. Larvae were considered infected if they expressed dark pigmentation (melanization) after inoculation. Larvae were scored as dead when they did not respond to touch stimuli by sterile tweezers. At the end of the experiments all larvae (dead or alive) were anesthetized on ice in order to minimize suffering and then killed at −80°C.

### Genomic and Proteomic Analysis of a Selected *Pseudomonas* spp. With Distinctive Phenotypic Traits

*Pseudomonas fluorescens* ITEM 17298, which exhibited a biofilm biomass higher than 2.0 (as CV Abs_570 nm_), significant differences in motility colony diameters, and the highest EPS production, was selected to be used in genomic and proteomic analysis.

#### Genomic Analysis

Genomic analysis of the selected strains was performed based on the draft genome sequence published in Fanelli et al. (2017). All the gene and protein sequences used in this study were retrieved from GenBank (NCBI). The homology-based relationship of the selected *Pseudomonas* spp. strain toward selected proteins was determined by BLASTP algorithm on the NCBI site^1^. Gene models were manually determined, and clustering and orientation were subsequently deduced for the closely linked genes.

In order to identify proteins involved in specific pathways (i.e. pyoverdine, pyomelanin and biofilm), we combined and compared the results of (i) search for specific domains in the UniProt KB list; (ii) proteome from selected group of proteins with specific features by using the Proteome comparison tool within PATRIC 3.5.43 (Wattam et al., 2017); and (iii) mapping UniProtKB AC/ID and protein homology-based search against the *Pseudomonas* database (Winsor et al., 2016).

For proteases identification proteins predicted with PFAM protease-associated domains were functionally annotated with InterPro© 76.0 (EMBL-EBI; Mitchell et al., 2019). Additionally, homology-based relationship was determined by MEROPS Blast service (Rawlings et al., 2018).

Virulence genes were identified by using VF analyzer pipeline implemented in the VFDB database (Liu et al., 2019). The automatic analysis was then manually confirmed and improved by homology search and functional prediction using InterProScan© 76.0.

#### Ge-LC/MS

Bacteria cells, from tree independent overnight cultures of treated or untreated strain, were inoculated (*ca.* 3 log cfu/mL), in multiwell plates containing 5 mL of M63 and incubated at 15°C for 72 h. The treatment with 0.75 mg/mL LFH, previously demonstrated as antimicrobial and antibiofilm agent (Quintieri et al., 2012, 2019b), was also included. The assayed concentration was selected, in a preliminary test, among decreasing LFH concentration values (3, 1.5, and 0.75 mg/mL), for its ability to reduce biofilm biomass more than 50% without affecting microbial load; bacterial growth, CFU counts were checked at 7, 24, 48, and 72 h of incubation by plating serial 10-fold dilutions on LB agar; plates were incubated at 30°C for 24 h.

After 72 h of incubation, planktonic cells were transferred to 15 mL Falcon tubes and harvested by centrifugation at *8500* × rpm for 10 min at 4°C; by contrast, biofilm biomass was quantified in multiwell plates as described above. Lysis of planktonic cells was carried out as reported by Quintieri et al. (2019b); after removing cell debris, soluble proteins were precipitated overnight at −20°C, by adding 6 volumes of ice-cold acetone, and resuspended in 8 M urea, 2 M thiourea buffer. Protein concentration was measured by Roti-Nanoquant (Carl Roth, GmbH, Germany), and 25 μg from each sample was separated by 1D-SDS-PAGE using Criterion TGX Precast Gels (BioRad Laboratories, Hercules, CA, United States) for 1 h at 150 V. Lanes were cut in ten equidistant pieces and subjected to trypsin in-gel digestion as described by Grube et al. (2015). The peptide mixture within each gel piece was desalted by Zip-Tip μC18 pipette tips (Millipore, United States) as previously reported (Quintieri et al., 2019b). LC-MS/MS analyses were done using an EASY-nLC coupled to a LTQ Orbitrap mass spectrometer equipped with nanoelectrospray ion source (Thermo Fisher Scientific, Waltham, MA, United States). The mass spectrometer was operated in positive ion mode. Peptides were directly loaded on a self-packed column (20 cm) with integrated emitter with a flow rate of 500 nL/min using buffer A (0.1% acetic acid). Peptides were separated by a 75 min non-linear gradient from 5 to 75% buffer B (0.1% acetic acid in acetonitrile) and a flow rate of 300 nL/min. All samples were measured in parallel mode; survey scans were recorded in the Orbitrap with a resolution of 30,000 in the mass range of 300 – 2000 m/z; the five most intensive peaks per scan cycle were selected for fragmentation in the LTQ. Precursor ions were dynamically excluded from fragmentation for 30 s; single-charged ions and ions with unknown charge state were excluded from fragmentation. Internal calibration was applied (lock mass 445.120025).

#### Protein Identification

For protein identification, spectra were searched against the annotated protein sequences from the respective *P. fluorescens* ITEM 17298 genome, including reverse sequences and common laboratory contaminants (11,526 entries; Fanelli et al., 2017). Database searches were performed using Sorcerer SEQUEST (Version v. 27 rev. 11, Thermo Fisher Scientific; Lundgren et al., 2009) and Scaffold 4.0.5 (Proteome Software, Portland, OR, United States) with the following search parameters: parent ion tolerance = 10 ppm, fragment ion mass tolerance = 1.00 Da, up to two missed cleavages allowed and methionine oxidation (+16 Da) set as variable modification (López-Mondéjar et al., 2016). Protein identifications were accepted if they contained at least 2 identified peptides. Protein quantification was based on the NSAF (Zybailov et al., 2006). Functional classification of proteins was done using Prophane 2.0^2^ and it is based ONIGRFAMs annotations. Voronoi treemaps were generated using Paver (Decodon, Greifswald, Germany)^3^. An analysis of KEGG pathways was also carried out; KO identifiers were extrapolated by Uniprot database through Uniprot accession numbers retrieved from genomic annotation.

### Statistical Analysis

After assessing homogeneity variance by Levene’s test (*P* < 0.05) a two-ANOVA was carried out using IBM SPSS Statistics 21.0 (SPSS, IBM corp., Armonk, NY, United States) to examine the effect time on biofilm biomass, swarming, swimming and twitching produced by each assayed strain. Multiple comparisons among individual means for each assayed strain were performed by Fisher’s least significant difference (LSD) multiple range test at the 95% confidence interval. When required, one-way ANOVA was performed followed by *post hoc* HSD Tukey test (*P* < 0.05). In the case of unequal variances, the non-parametric Kruskal–Wallis *H* test was applied with appropriate Games-Howell’s *post hoc* test.

Kaplan-Meier survival analysis was performed to study survival percentage of infected larvae. A log-rank test was run to determine if there were differences in the survival distribution for the different types of intervention.

Proteins detected in two out of three biological replicates were considered for statistical analysis using MeV v4.8.1 (Saeed et al., 2003). Each group of samples was compared by Student’s *t*-test with a *P*-value of 0.01. Only proteins showing at least twofold changes in addition statistical significance were considered for further analysis. So-called “OFF/ON” proteins needed to be detected or absent in at least two replicates of one experimental condition.

## Results

### Phenotypic Traits

Nine out of 19 strains, belonging to *P. fluorescens* and *P. gessardii* species, produced pigmented or fluorescent colonies on King B at 15°C (Supplementary Table S1); 2 out of 10 *P. fluorescens* strains also grew as brown colonies on King A and King B and only ITEM 17298 and ITEM 17299 produced dark pigment on M63 medium at the fourth day of incubation at 15°C. Among the remaining species, *P. gessardii* PS36 and 2A also produced pink colonies on King A (Supplementary Table S1).

As regards biofilm production, ITEM 17298 produced the highest biofilm biomass (on average, CV Abs_570 nm_ > 4.4 ± 0.23; Supplementary Figure S1). Interestingly, pseudomonads producing colored or fluorescent colonies on at least one selective medium registered a biofilm biomass amount higher (0.68 ± 0.041 < CV Abs_570 nm_ > 4.5 ± 0.2) than that produced by not pigmenting strains (CV Abs_570 nm_ < 0.33 ± 0.45) after 72 h of incubation. These latter included *P. fragi* strains (Supplementary Figure S1).

There was no statistically significant [*F*(36,114) = 0.709, *p* = 0.881] interaction between the effects of the assayed strains and time of incubation on biofilm biomass. Likewise, biofilm amount of each strain did not change significantly during assay (*P* > 0.05), whereas highly significant (*P* < 0.0001) differences in biofilm biomass were found among the assayed strains. However, as shown in the Supplementary Figure S1, only the strains ITEM 17298, ITEM 17299, PS36, and NCPPB 1964 reached average biomass values ranking between 4.50 and 2.42. Moderate and similar (*P* > 0.05) biofilm production occurred in the cultures of the strains 84094, PZ20, 2A, RC3, and RC4. On the contrary, a little more than appreciable biomass was produced by the remaining strains.

On the basis of these results, four strains (*P. fluorescens* ITEM 17298, ITEM 17299, NCPPB 1964, and *P. gessardii* PS36) producing biofilm biomass values higher than 2.0 were evaluated in motility assays and for EPS production. Two negative biofilm-former strains (*P. fragi* PS4 and *P. fluorescens* PS37) were also included. Results regarding the quantification of EPS by Congo Red partially agreed with biofilm-related data. Indeed, concentrations of 37 and 23 μg/mL of bound Congo Red (y_OD__490 nm_ = 0.040 x_EPS μg/mL_ + 0.039; *R*^2^ = 0.991) were determined for ITEM 17298 and ITEM 17299, respectively. *P. fluorescens* NCPPB 1964 produced 9.4 μg/mL, whilst the remaining strains registered EPS values lower than 3.4 μg/mL (Supplementary Table S2). EPS production was also showed in agar plates (Supplementary Figure S2). As concerns motility, swarming, swimming and twitching, they were evaluated on the same selected strains (Supplementary Figure S3).

All strains were unable to swarm at 15°C in the assayed period; indeed, *Pseudomonas* spp. colonies showed only slight increases in their diameter over the time (4.72 ± 0.23 mm and 6.61 ± 0.29 mm; Supplementary Figure S3A) and no appearance of elongated swarm edge (Supplementary Figure S4). By contrast, they shared increases in colony diameter, displayed in the swimming assay, suggested that this kind of motility was favored for all selected strains in experimental condition (Supplementary Figure S3B); in addition, ITEM 17299 almost doubled its diameter in comparison to the other strains after 72 h of incubation.

As concerns twitching, *P. fluorescens* ITEM 17298 exhibited this kind of motility at 48 h and reached the highest value of absorbance (CV_570 nm_) after additional 24 h of incubation. Among the remaining strains, only two strains (*P. fluorescens* 1964 and ITEM 17299) registered a moderate twitching motility determined by absorbance values higher than 0.2 (Supplementary Figure S3C). In light of these results, *P. fluorescens* ITEM 17298, exhibiting distinctive phenotypic traits, was selected to be investigated by genomic and proteomic analysis.

### Minimal Biofilm Inhibitory Concentration (MBIC) of HLF

Supplementary Figure S5A shows the effect of the assayed HLF concentration on ITEM 17298 load. Starting from 24 h of incubation, decrements in counts by 2.1 ± 0.15 and 1.3 ± 0.2 log cfu/mL were registered at 3 and 1.5 mg/mL of HLF (*P* = 1.109 × 10^–7^), respectively; the inhibitory effect persisted for 72 h. By contrast, no significant differences in counts occurred, between the cultures supplemented by 0.75 mg/mL of HLF and the untreated samples throughout the incubation (Supplementary Figure S5A).

As regards biofilm, a statistically significant interaction between the effects of time and HLF level on biomass increments [*F*(8, 60) = 40.284, *p* = 1 × 10^–30^] was found. Moreover, simple main effects analysis showed that the addition of HLF caused a highly significant (*p* < 0.0001) dose-response reduction of biofilm biomass starting from 24 h of incubation. By contrast, the untreated sample showed a significant biofilm biomass increase (0.24 units, on average; *p* = 0.030) already in the early stages of the experiment. After 72 h of incubation the lowest HLF concentration reduced biofilm biomass by *ca.* 62% (Supplementary Figure S5B). Thus, the HLF level of 0.75 mg/mL was, then, selected for comparative proteomic analysis. The absence of discoloration was also found in all treated samples in comparison to the untreated ones (Supplementary Figure S5C).

### Proteomic Analysis of *P. fluorescens* ITEM 17298 Under HLF Treatment

**FIGURE 1:**
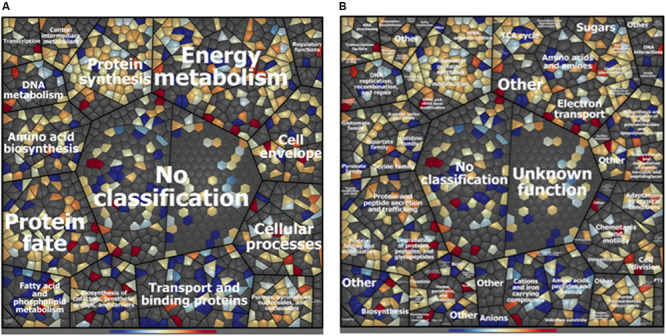
Voronoi treemap visualization of *P. fluorescens* ITEM 17298 protein patterns after treatment with 0.75 mg/mL of HLF in M63 at 15°C for 72 h. Proteins are depicted as single cells and grouped according to their functional classification. Classification was achieved using Prophane 2.0 software and is based on TIGRFAMs. The classification level main role **(A)**, subrole **(B)** are depicted. Proteins with higher amounts in treated cells are shown in blue; proteins with higher amounts in untreated cells are shown in red. Gray cells represent proteins that were not identified in the experimental conditions.

A total of 993 cytoplasmic and cell surface proteins were identified in ITEM 17298 proteome after 72 h of HLF treatment. Among these, 27 and 103 were found to be exclusively expressed in treated or untreated samples, respectively, whereas, based on fold-change calculation (>twofold), 94 proteins were found to be differentially expressed (Table 1 and Supplementary Table S3). Treatment affected protein expression across all functional categories, especially those related to transport and binding, and fatty acid and phospholipid metabolism (Figure 1); *ca* 22% of the identified proteins were without functional classification (Supplementary Table S3).

**TABLE 1:** Fold changes of *P. fluorescens* ITEM 17298 proteins synthesized in the treated samples in comparison to the untreated ones (≥two fold, *P* < 0.05).

Identifier	Function	Fold change T/UT
*Amino acid biosynthesis*		
PROKKA_03771	Acetolactate synthase isozyme 3 small subunit	OFF^1^
PROKKA_02986	Imidazoleglycerol-phosphate dehydratase	OFF
PROKKA_02503	L-lysine cyclodeaminase	OFF
PROKKA_01540	Sulfite reductase [NADPH] flavoprotein alpha-component	OFF
PROKKA_01465	2-isopropylmalate synthase	2.36
PROKKA_00590	Shikimate dehydrogenase	2.11
PROKKA_03075	3-dehydroquinate synthase	OFF
PROKKA_01520	Histidinol-phosphate aminotransferase 2	OFF
PROKKA_02584	Hydroxymethylglutaryl-CoA lyase YngG	OFF
PROKKA_03377	Diaminopimelate decarboxylase	3.25
PROKKA_05608	Ornithine carbamoyltransferase, catabolic	2.16
*Biosynthesis of cofactors, prosthetic groups, and carriers*		
PROKKA_03339	Coenzyme A biosynthesis bifunctional protein CoaBC	ON^2^
PROKKA_03089	p-hydroxybenzoate hydroxylase	ON
PROKKA_00435	Thiazole synthase	ON
PROKKA_02509	2-oxoisovalerate dehydrogenase subunit beta	OFF
PROKKA_05499	ATP-dependent dethiobiotin synthetase BioD 1	OFF
PROKKA_05004	Gamma-glutamyltranspeptidase precursor	OFF
PROKKA_02355	tRNA-modifying protein YgfZ	OFF
PROKKA_04172	Putative metal chaperone YciC	OFF
PROKKA_03405	Delta-aminolevulinic acid dehydratase	4.34
*Cell envelope*		
PROKKA_01908	Membrane bound L-sorbosone dehydrogenase	ON
PROKKA_02411	Membrane-bound lytic murein transglycosylase A precursor	ON
PROKKA_04757	Acyl-[acyl-carrier-protein]-UDP-N-acetylglucosamine O- acyltransferase	OFF
PROKKA_02911	D-methionine-binding lipoprotein MetQ precursor	OFF
PROKKA_01061	Lipopolysaccharide exporter periplasmic protein	OFF
PROKKA_00908	UDP-N-acetyl-D-glucosamine 6-dehydrogenase	OFF
PROKKA_03138	Bifunctional protein HldE	3.31
PROKKA_03604		
*Cellular processes*		
PROKKA_01123	Cell division protein FtsA	ON
PROKKA_04562	Filamentous hemagglutinin	ON
PROKKA_05339	Methyl-accepting chemotaxis protein McpS	ON
PROKKA_00251	Acyl-homoserine lactone acylase PvdQ precursor	OFF
PROKKA_05595	Hemolysin precursor	OFF
PROKKA_01052	Superoxide dismutase [Mn/Fe]	OFF
PROKKA_00543	Catalase HPII	3.31
PROKKA_01124	Cell division protein FtsZ	4.74
*Central intermediary metabolism*		
PROKKA_03207	Succinate-semialdehyde dehydrogenase [NADP(+)]	OFF
PROKKA_03275	Biosynthetic arginine decarboxylase	2.23
*DNA metabolism*		
PROKKA_04579	Putative type I restriction enzymeP M protein	ON
PROKKA_00722	DNA-binding protein HU-beta	OFF
PROKKA_02589	Exodeoxyribonuclease III	OFF
PROKKA_03903	ATP-dependent RNA helicase DeaD	5.17
PROKKA_03707	Type I restriction enzyme EcoKI M protein	3.13
PROKKA_04737	Cold shock protein CapB	OFF
*Energy metabolism*		
PROKKA_00359	Formate-dependent nitrite reductase complex subunit NrfG	ON
PROKKA_01246	L-lactate dehydrogenase [cytochrome]	ON
PROKKA_00959	NADH dehydrogenase	ON
PROKKA_02634	NADH-quinone oxidoreductase subunit F	ON
PROKKA_00268	Glutathione S-transferase GstB	OFF
PROKKA_01079	Malate:quinone oxidoreductase	OFF
PROKKA_02581	Methylmalonyl-CoA carboxyltransferase 12S subunit	OFF
PROKKA_04798	Phosphoenolpyruvate carboxylase	OFF
PROKKA_01666	Ureidoglycolate lyase	OFF
PROKKA_00562	Gluconate 2-dehydrogenase flavoprotein precursor	2.02
PROKKA_02238	Glyceraldehyde-3-phosphate dehydrogenase	3.97
PROKKA_02510	2-oxoisovalerate dehydrogenase subunit alpha	OFF
PROKKA_01175	3-hydroxyisobutyrate dehydrogenase	OFF
PROKKA_01050	Fumarate hydratase class II	OFF
PROKKA_02580	Hydroxycinnamoyl-CoA hydratase-lyase	OFF
PROKKA_03027	Imidazolonepropionase	OFF
PROKKA_02508	Lipoamide acyltransferase component of branched-chain alpha-keto acid dehydrogenase complex	OFF
PROKKA_02048	Methylisocitrate lyase	OFF
PROKKA_04188	NADPH azoreductase	OFF
PROKKA_05277	Oxaloacetate decarboxylase	OFF
PROKKA_01886	Succinylglutamate desuccinylase	OFF
PROKKA_03013	Fructose-1,6-bisphosphatase class 1	2.35
PROKKA_04273	Glucose-6-phosphate 1-dehydrogenase	2.59
PROKKA_00052	Periplasmic beta-glucosidase precursor	2.56
PROKKA_02836	UDP-glucose 4-epimerase	0.27
PROKKA_02382	Alcohol dehydrogenase 2	2.11
PROKKA_05609	Arginine deiminase	2.51
PROKKA_00695	Aspartate ammonia-lyase	2.70
*Fatty acid and phospholipid metabolism*		
PROKKA_01847	3-oxoacyl-[acyl-carrier-protein] reductase FabG	ON
PROKKA_05080	Malonyl CoA-acyl carrier protein transacylase	ON
PROKKA_00677	2-oxoglutarate carboxylase small subunit	OFF
PROKKA_01444	Acetyl-CoA acetyltransferase	OFF
PROKKA_05221	Acyl-CoA dehydrogenase	OFF
PROKKA_05219	Acyl-CoA dehydrogenase	OFF
PROKKA_05661	Long-chain-fatty-acid–CoA ligase	OFF
PROKKA_01442	Putative succinyl-CoA:3-ketoacid coenzyme A transferase subunit A	OFF
PROKKA_02046	2-methylcitrate synthase	OFF
PROKKA_04758	3-hydroxyacyl-[acyl-carrier-protein] dehydratase FabZ	OFF
PROKKA_05218	Acetyl-CoA acetyltransferase	OFF
PROKKA_02045	Aconitate hydratase 1	OFF
PROKKA_02582	Acyl-CoA dehydrogenase	OFF
PROKKA_05217	3-oxoacyl-[acyl-carrier-protein] reductase FabG	0.14
PROKKA_01830	C-factor	0.50
*No classification*		
PROKKA_03843	Bacteriophage N4 receptor, outer membrane subunit	ON
PROKKA_03861	Dehydrosqualene desaturase	ON
PROKKA_05325	General stress protein 69	ON
PROKKA_03224	Putative assembly protein	ON
PROKKA_00705	Putative glucose-6-phosphate 1-epimerase	ON
PROKKA_01433	Transglutaminase-like superfamily protein	ON
PROKKA_00175	Carboxymethylenebutenolidase	OFF
PROKKA_03131	Decarbamoylnovobiocin carbamoyltransferase	OFF
PROKKA_05205	Hypothetical protein	OFF
PROKKA_03689	Hypothetical protein	OFF
PROKKA_04718	Hypothetical protein	OFF
PROKKA_02569	Putative chaperone protein EcpD	OFF
PROKKA_00373	Putative lipoprotein YgdR precursor	OFF
PROKKA_01786	Putative pterin-4-alpha-carbinolamine dehydratase	OFF
PROKKA_03426	Quinone oxidoreductase 1	OFF
PROKKA_04378	SCP-2 sterol transfer family protein	OFF
PROKKA_00834	Serralysin precursor	OFF
PROKKA_01563	Carboxymuconolactone decarboxylase family protein	OFF
PROKKA_00144	Cupin domain protein	OFF
PROKKA_02708	Hypothetical protein	OFF
PROKKA_05173	Hypothetical protein	OFF
PROKKA_00895	Indole-3-glycerol phosphate synthase	OFF
PROKKA_05375	Membrane dipeptidase (Peptidase family M19)	OFF
PROKKA_03324	NAD dependent epimerase/dehydratase family protein	OFF
PROKKA_02300	NmrA-like family protein	OFF
PROKKA_01002	Porin D precursor	OFF
PROKKA_01096	Hypothetical protein	0.25
PROKKA_05590	Hypothetical protein	2.38
PROKKA_00069	N-ethylmaleimide reductase	3.13
PROKKA_05649	Porin B precursor	0.38
PROKKA_04692	PrkA AAA domain protein	0.22
PROKKA_05645	Putative sugar-binding periplasmic protein precursor	0.37
*Protein fate*		
PROKKA_01230	Glutamyl aminopeptidase	ON
PROKKA_01048	Peptidase PmbA	ON
PROKKA_04376	Putative protease SohB	ON
PROKKA_03301	Hypothetical protein	OFF
PROKKA_04770	Methionine aminopeptidase	OFF
PROKKA_02401	Leukotoxin	2.75
PROKKA_02543	ATP-dependent Clp protease proteolytic subunit	OFF
PROKKA_05384	Carboxypeptidase G2 precursor	OFF
PROKKA_01697	Extracellular serine protease precursor	OFF
PROKKA_03117	Peptide methionine sulfoxide reductase MsrA	OFF
PROKKA_02089	putative lipoprotein YiaD precursor	2.54
PROKKA_04761	Outer membrane protein assembly factor BamA precursor	2.28
PROKKA_01469	Outer membrane protein assembly factor BamB precursor	2.33
*Protein synthesis*		
PROKKA_01492	Queuine tRNA-ribosyltransferase	ON
PROKKA_04982	30S ribosomal protein S16	OFF
PROKKA_03573	Acetyltransferase (GNAT) family protein	OFF
PROKKA_01468	GTPase Der	OFF
PROKKA_02661	Serine–tRNA ligase	OFF
PROKKA_03741	tRNA pseudouridine synthase B	2.19
PROKKA_00026	30S ribosomal protein S19	OFF
PROKKA_00045	30S ribosomal protein S4	3.53
*Purines, pyrimidines, nucleosides, and nucleotides*		
PROKKA_03839	Ribose-phosphate pyrophosphokinase	2.46
PROKKA_03262	Bifunctional purine biosynthesis protein PurH	2.15
*Regulatory functions*		
PROKKA_00978	Catabolite repressor/activator	OFF
PROKKA_01026	HTH-type transcriptional regulator LutR	OFF
PROKKA_00527	Alginate biosynthesis transcriptional regulatory protein AlgB	5.156
*Transcription*		
PROKKA_04367	Transcriptional regulator LsrR	ON
*Transport and binding proteins*		
PROKKA_01304	Fatty acyl-CoA reductase	ON
PROKKA_00683	Cystine-binding periplasmic protein precursor	OFF
PROKKA_04296	D-galactose-binding periplasmic protein precursor	OFF
PROKKA_05370	Ferripyoverdine receptor precursor	OFF
PROKKA_02443	Glycine betaine-binding protein OpuAC precursor	OFF
PROKKA_04650	Hemin receptor precursor	OFF
PROKKA_00538	High-affinity zinc uptake system protein ZnuA precursor	OFF
PROKKA_05330	Hypothetical protein	OFF
PROKKA_01003	Periplasmic dipeptide transport protein precursor	OFF
PROKKA_00656	Putative periplasmic iron-binding protein precursor	OFF
PROKKA_01603	Putative TonB-dependent receptor precursor	OFF
PROKKA_05646	sn-glycerol-3-phosphate transport system permease protein UgpA	OFF
PROKKA_05019	LPS O-antigen length regulator	2.62
PROKKA_02316	Bacterial extracellular solute-binding protein	OFF
PROKKA_01319	Hemin-binding periplasmic protein HmuT precursor	OFF
PROKKA_03761	Hemin-binding periplasmic protein HmuT precursor	OFF
PROKKA_04599	hypothetical protein	OFF
PROKKA_03209	Leucine-, isoleucine-, valine-, threonine-, and alanine-binding protein precursor	OFF
PROKKA_01352	Lysine-arginine-ornithine-binding periplasmic protein precursor	OFF
PROKKA_04728	Lysine-arginine-ornithine-binding periplasmic protein precursor	OFF
PROKKA_01001	Periplasmic dipeptide transport protein precursor	OFF
PROKKA_04286	Putative TonB-dependent receptor BfrD precursor	OFF
PROKKA_00355	Sulfate-binding protein precursor	OFF
PROKKA_02788	D-ribose-binding periplasmic protein precursor	0.43
PROKKA_05029	Iron uptake system component EfeO precursor	0.27
PROKKA_00101	Leucine-, isoleucine-, valine-, threonine-, and alanine-binding protein precursor	0.36
PROKKA_01004	Periplasmic dipeptide transport protein precursor	0.42
PROKKA_03225	Bacterioferritin	3.82

*^1^OFF exclusively detected in untreated samples. ^2^ON exclusively detected in treated samples.*

**FIGURE 2:**
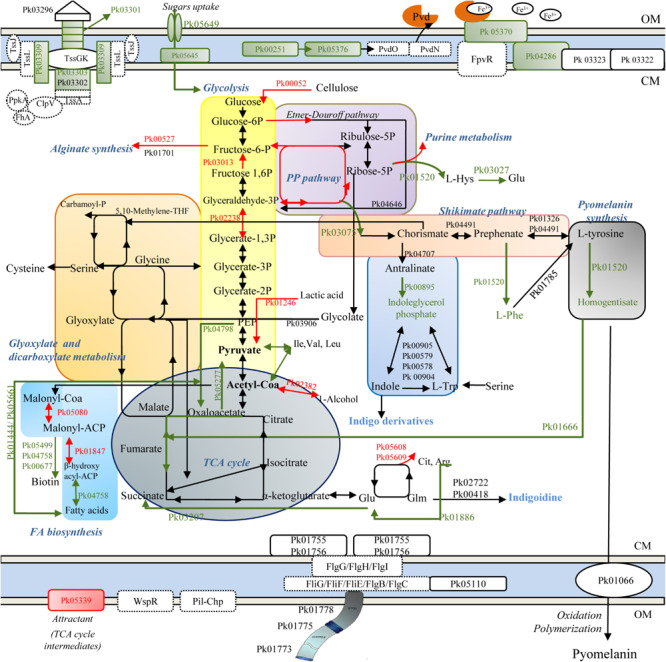
Schematic representation of metabolic pathways in ITEM 17298, obtained by genomic (dashed line) and proteomic analysis (solid line). Colored arrows and squares indicate induced (red lines) or repressed (green) pathways under HLF treatment in comparison to metabolic pathways detected in untreated cells. Pk03296, type VI secretion system Vgr family protein; Pk03301, type VI secretion system effector, Hcp1 family; Pk03302, type VI secretion protein; Pk03303, type VI secretion protein; Pk03309, type VI secretion protein IcmF; TssK, hypothetical protein type IV secretion system baseplate subunit TssK; TssG, hypothetical protein type IV secretion system baseplate subunit TssG; TssL, type IV secretion system protein TssL, TssA, hypothetical protein type Iv secretion system protein TssA; ClpV, Chaperone protein ClpB; PpkA, Serine/threonine-protein kinase PrkC FhA, FHA domain protein; Pvd, pyoverdine; Pk00251, 3-oxo-C12-homoserine lactone acylase PvdQ; Pk05376, pyoverdine biosynthesis protein PvdP; PvdO, pyoverdine biosynthesis protein PvdO; PvdN, Pyoverdin biosynthesis protein PvdN, putative aminotransferase, class V; Pk05370, ferripyoverdine receptor; Pk04286, TonB-dependent siderophore receptor; Pk0322, TonB system transport protein ExbD; Pk0323 TonB-system energizer ExbB; Pk00052, Periplasmic beta-glucosidase precursor; Pk00527, Alginate biosynthesis transcriptional regulatory protein AlgB; Pk01701, GDP-mannose 6-dehydrogenase; Pk01520, deoxyuridine 5′-triphosphate nucleotidohydrolase; Pk04646, phosphogluconate dehydratase; Pk04491, prephenate dehydratase; Pk01326, aromatic-amino-acid aminotransferase; Pk04707, Anthranilate synthase component 1; Pk01520, Histidinol-phosphate aminotransferase 2; Pk01785, phenylalanine-4-hydroxylase; Pk00895, Indole-3-glycerol phosphate synthase; Pk03075, 3-dehydroquinate synthase; Pk00905, Tryptophan synthase alpha chain; Pk00579, Tryptophan synthase alpha chain; Pk00578, Tryptophan synthase beta chain; Pk 00904, Tryptophan synthase beta chain; Pk01666, fumaryl acetoacetase; Pk05608, ornithine carbamoyltransferase; Pk05609, arginine deiminase; Pk02722, Tyrocidine synthase 3; Pk00418, phosphopantetheine adenylyltransferase; Pk04798, Phosphoenolpyruvate carboxylase; Pk01246, L-lactate dehydrogenase; Pk03906, Phospho-2-dehydro-3-deoxyheptonate aldolase; Pk02382, Alcohol dehydrogenase 2; Pk05277, Oxaloacetate decarboxylase; Pk03207, Succinate-semialdehyde dehydrogenase; Pk03013, Fructose-1,6-bisphosphatase class 1; Pk02238, Glyceraldehyde-3-phosphate dehydrogenase; Pko1444, Acetyl-CoA acetyltransferase; Pk05661, Long-chain-fatty-acid–CoA ligase; Pk05499, ATP-dependent dethiobiotin synthetase BioD 1; Pk04758, 3-hydroxyacyl-[acyl-carrier-protein] dehydratase FabZ; Pk00677, 2-oxoglutarate carboxylase small subunit; Pk05080, Malonyl CoA-acyl carrier protein transacylase; Pk01847, 3-oxoacyl-[acyl-carrier-protein] reductase FabG; Pk01066, phospholipid ABC transporter-binding protein MlaD; Pk01755, Flagellar motor switch protein FliN; Pk01756, Flagellar motor switch protein FliM; Pk05110, Flagellar brake protein YcgR; Pk01773, Flagellar hook-associated protein 2; Pk01775, B-type flagellin; Pk01778, Flagellar hook-associated protein 1; WspR, two-component response regulator; FlgG, Flagellar motor switch protein FliG; FliH, Flagellar assembly protein FliH; FliI, Flagellum-specific ATP synthase; FliF, Flagellar M-ring protein, FliE, Flagellar hook-basal body complex protein FliE; FlgC, B-type flagellin; FlgB, Flagellar basal body rod protein FlgB; Pil-Chp, Type IV pili twitching motility related proteins. OM, outer membrane; CM, cytoplasmic membrane.

A depiction of major cellular pathways, identified by integrating genomic and proteomic results and affected or not by the treatment, is showed in Figure 2. In particular, HLF treatment caused a reduction in the levels of transport and binding proteins; these latter included ABC transporter and periplasmic proteins involved in the uptake of amino acids (PROKKA_01002; PROKKA_01352; and PROKKA_02911), osmoprotectant (PROKKA_02443), anions (sulfate and phosphate), cations (nickel ABC transporter), and iron (PROKKA_00355, PROKKA_05330, PROKKA_01319, PROKKA_03761; and PROKKA_05029), carbohydrates and sugars (such as ribose, glucose, mannitol, fructose and glycerol; PROKKA_02788; PROKKA_05645, and PROKKA_05649) (Table 1). Other membrane components, negatively affected by HLF, were surface-sensing chemotaxis systems toward sugars and dipeptides (PROKKA_04296, PROKKA_01001, and PROKKA_01003). By contrast, the treatment increased the amount of PROKKA_05339, a specific chemoreceptor for tricarboxylic acid (TCA) cycle intermediates (butyrate and acetate).

As consequence of the reduced sugar and carbohydrate uptakes, glycolysis was putatively supplied by pentose phosphate intermediates, synthesized by the related HLF induced enzymes, and by carbohydrate metabolism (Table 1). As concern TCA cycle, HLF reduced the amounts of enzymes related to the conversion from fumarate to oxaloacetate (PROKKA_01050 and PROKKA_01079), precursor for the synthesis of amino acids and citrate.

Increased levels of PROKKA_02382 together with PROKKA_00959 and PROKKA_02634 (Table 1) were also found under HLF treatment suggesting a their possible role in NADH/NAD^+^ ratio restoring and the respiratory chain support to avoid the shift into non-growing conditions.

Proteins associated with pyomelanin synthesis were revealed by *in silico* prediction and also detected in ITEM 17298 proteome (Supplementary Tables S3, S4 and Figure 2). These latter included proteins involved in pyrimidine biosynthesis (PROKKA_03334, PROKKA_04036, and PROKKA_02015), and membrane proteins (PROKKA_01067 and PROKKA_01066). As depicted in Figure 2, the biosynthesis of most proteins correlated to pyomelanin pathway was inhibited by the treatment. In particular, in HLF treated cells we found reduced levels of enzymes responsible for the conversion of L-tyrosine to homogentisate, precursor of pyomelanin in the extracellular environment. Reduction in homogentisate amount also hinders the TCA cycle maintenance by the conversion to the related intermediates (Figure 2). Pyomelanin synthesis crosses the shikimate pathway, producing chorismate from D-erythrose 4-phosphate, a pentose phosphate intermediate; both the enzyme related to this latter reaction (PROKKA_03075), as well the indoleglycerol phosphate (PROKKA_00895), involved in the synthesis of indigo derivates from tryptophan, were inhibited by the treatment.

In accordance with the reduction of iron transporter levels, proteins related to the synthesis of fluorescent siderophores (pyoverdines, Pvds) (PROKKA_01890, PROKKA_05531, PROKKA_04061, PROKKA_00251, and PROKKA_05376) and their signaling cascade (TonB receptor-like PROKKA_01603 and PROKKA_04286, the ferripyoverdine receptor PROKKA_05370 and the hemin receptor PROKKA_04650) were also negatively affected by HLF. A list of all the proteins involved in pyoverdine metabolism in ITEM 17298 is also reported in Supplementary Table S5.

Several proteins with regulatory functions were identified in cold stored cells (PROKKA_00457, PROKKA_00527, PROKKA_00726, PROKKA_00978, PROKKA_01793 PROKKA_01987, and PROKKA_05362). Among these the fructose operon transcriptional repressor (PROKKA_00978) and HTH-type transcriptional regulator LutR (PROKKA_01026), involved in the regulation of carbon metabolism and L-lactate utilization, respectively, were inhibited by HLF. By contrast, the alginate biosynthesis transcriptional regulatory protein AlgB (PROKKA_00527) and the transcriptional regulator LsrR (PROKKA_04367), involved in alginate biosynthesis and biofilm formation under the regulation of QS, were induced by the treatment.

In ITEM 17298, type IV secretion system (also named T6SS) was detected by genomic and proteomic analysis (genomic locus PROKKA_03297-03312; Figure 2). The structural organization in bacterial cell wall of T6SS secretion system is depicted in Figure 2 and consists of TssB (PROKKA_03303), TssC (PROKKA_03302), Hcp (hemolysin co-regulated protein, PROKKA_03301), and VgrG (valine-glycine repeat protein G, PROKKA_03297). In the treated cell, T6SS showed reduced amounts of some constituting proteins (Figure 2 and Table 1).

Type I secretion system (TISS), composed of a tripartite complex formed by an ABC-transporter, a membrane fusion protein (PROKKA_04534), and a TolC-like outer membrane protein (PROKKA_04532) were also detected in ITEM 17298. A putative effector of TISS was PROKKA_00834, the alkaline protease AprA, only detected in untreated cells (Table 1).

Other proteins correlated with pathogenesis were detected in ITEM 17298; in particular, the effector hemolysin precursor (SlhA, PROKKA_05595) was repressed by HLF, whilst the signal peptidase I (LepB, PROKKA_01619), and the esterase EstA precursor (PROKKA_04708), did not change their amount.

### Determination of *P. fluorescens* Infection in *Galleria mellonella* Model

**FIGURE 3:**
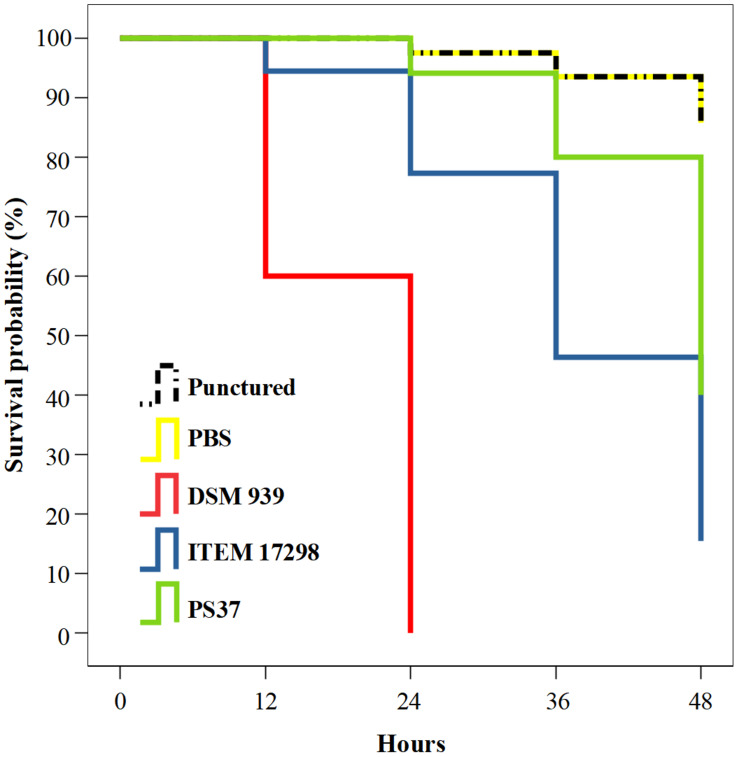
Kaplan-Meier plot showing percentage survival of *Galleria mellonella* larvae after inoculation with PBS bacterial suspensions of either *Pseudomonas fluorescens* ITEM 17298, PS37 or *P. aeruginosa* DSM 939. Mortality of not infected control larvae are inoculated with sterile PBS or simply injured with syringe needle (punctured). For each treatment *n* = 14 (pooled from duplicate experiment).

The infected larvae showed a treatment-dependent estimated survival mean time. In particular, 45 and 38 days of survival are estimated for larvae infected with cold adapted cells of *P. fluorescens* strains PS37 and ITEM 17298, respectively; *ca.*50% of the latter subjects died during the first 36 h. By contrast, median survival time of larvae inoculated with *P. aeruginosa* DSM 939 was 24 h, as previously reported (Hill et al., 2014). The simple puncture with syringe needle or inoculation with the sterile diluent caused a very low mortality of larvae (7.72% on average starting from 24 to 48 h of incubation). The survival distributions for the five treatments were statistically different according to a log rank test [$\chi_{\left(4\right)}^{2}$ = 135.624, *p* < 0.00001]. The Kaplan-Meier plot in Figure 3 shows that the survival probability is lower for larvae infected with ITEM 17298 in comparison with those inoculated with PS37 from 12 h to the end of the assay. Overall, the mortality percentages of larvae infected with ITEM 17298 and PS37 were 66.7 and 50%. The hazard ratio (estimating how rapidly the risk of death event occurs) in larvae infected by ITEM 17298 was 2.2741 times higher than that inoculated with PS37 (95% CI), but similar (*P* = 0.2324) to that of larvae treated with *P. aeruginosa* DSM 939.

## Discussion

Psychrotrophic food spoilage pseudomonads show a range of characteristics that favor their persistence and adaptation under prohibitive conditions for other microbial groups. In this work most assayed strains, previously isolated from cold stored cheeses, showed at low temperature a wide pattern of pigments, higher biofilm and EPS amounts and the appearance of motility elements in comparison to the same strains cultured at higher temperature. This adaptive response was investigated by means proteomic and genomic analyses of ITEM 17298 chosen as model for its distinctive phenotypic traits. After checking of concentration value able to inhibit biofilm without affecting the growth of bacteria in the assayed medium, the application of sub-lethal concentration of HLF allowed to reveal major metabolic pathways. Thus, HLF confirmed its antibiofilm activity under novel experimental conditions.

Although *P. fluorescens* ITEM 17298 cannot be considered a pathogen as *P. aeruginosa*, rapidly causing the death of larvae, preliminary results from *G. mellonella* assay showed, for the first time, that this spoiler had a moderate pathogenic potential with possible implications in food safety risk assessment. These results were also supported by genomic and proteomic analysis as reported above.

### Proteins Involved in Biofilm Formation

In accordance with the results from motility assay, the occurrence of genes related to surface-sensing Wps (PROKKA_04814-PROKKA_04820) Pil-Chp (PROKKA_00456-PROKKA_00461) systems were firstly revealed in ITEM 17298. Pil-Chp system, includes a cascade of intracellular signaling molecules responsible for Type IV pili production (Armbruster and Parsek, 2018) and putatively it could be involved in ITEM 17298 twitching motility (Supplementary Figure S3C). In addition, the response regulator PleD (PROKKA_00457), belonging to Pil-Chp system, was detected also in ITEM 17298 proteome. However, this latter did not change its amount under HLF treatment. This result agrees with our previous data (Quintieri et al., 2019b) and suggests that PleD activation is affected exclusively by cold incubation. By contrast, increased levels of Methyl-accepting chemotaxis protein M (PROKKA_05339) was registered in presence of HLF; McpS putatively provided TCA cycle intermediates as carbon and energy source (Lacal et al., 2011) due to a compromised TCA cycle and a reduced sugars and carbohydrates uptake (Figure 2).

Both Wps and Pil-Chp systems were regulated by nucleotide-based signals (c-di-GMP and cAMP) (Jimenez et al., 2012; Fazli et al., 2014). The regulation of cellular functions by c-di-GMP occurs by specific receptor at multiple levels (allosteric regulation of proteins, modulation of a transcription factor; direct interaction with non-coding RNA molecules (Valentini and Filloux, 2016). Several EPS, such as cellulose and alginate, are regulated by c-di-GMP, which serves as allosteric regulator of related synthetases.

The regulatory network of alginate biosynthesis is highly complex and occurs on different levels; in *P. aeruginosa* a total of 24 genes were identified (Franklin et al., 2011), that are all present in *P. fluorescens* ITEM 17298 genome, with the exception of the positive regulator of the alginate biosynthesis, *mucC*. The 12 genes responsible for the biosynthesis of the polysaccharide are located in an operon with the same content and organization of *P. aeruginosa* (Supplementary Figure S6), although flanking regions show differences in the genetic context.

Interestingly, in the treated ITEM 17298 levels of AlgB (PROKKA_00257) were increased; AlgB is a two-component system regulator responsible for the expression of AlgD (PROKKA_01701), promoter of alginate synthesis. Although the amount of biofilm in the treated cells was lower in comparison to the untreated, the synthesis of alginate could represent a resistance mechanism of bacteria to AMPs as reported for *P. aeruginosa* biofilm embedded cells (Yasir et al., 2018).

In accordance with our previous results (Quintieri et al., 2019b), HLF treatment repressed the synthesis of the HTH-type transcriptional regulator LutR. As previously discussed, LutR is part of the global complex signaling network governing several cellular processes, including biofilm formation (Quintieri et al., 2019b). In addition to LutR, HLF also negatively affected the synthesis of PROKKA_00978, the D-fructose-responsive transcription factor, also named glycolytic flux regulator Cra, for the first time identified in the proteome of this species. Lack of binding between Cra protein and its target promoters was found to strengthen the negative effect of glucose on fimbria curli production, during host colonization and biofilm formation (Rossi E. et al., 2018).

In ITEM 17298 type IV secretion system, also named T6SS, was detected by genomic and proteomic analysis (Figure 2). This bacterial component, forming a pathogenicity island in *P. aeruginosa* (Chen et al., 2015), will be also discussed as pathogenesis determinant in the subsequent paragraph. Recently, it has been demonstrated that the T6SS component Hcp (hemolysin co-regulated protein, PROKKA_03301) also takes part in biofilm formation in *P. aeruginosa* and *P. fluorescens*. In particular, during contact on a solid surface, Hcp affects pseudomonas mucoidy, motility and also exerts antibacterial activity with competitive bacteria (Decoin et al., 2015; Gallique et al., 2017). As shown in Table 1, and in accordance with biofilm biomass reduction, the synthesis of Hcp1 in ITEM 17298 was inhibited by HLF treatment. These results could be pivotal to deeper investigate biofilm inhibition by HLF, since the Hcp1 deletion in pathogenic pseudomonads caused an attenuation of biofilm-specific antibiotic resistance (Zhang et al., 2011).

### Proteins Involved in Pigment Synthesis

The results from pigmentation trials showed that ITEM 17298 produced at least two different pigments (fluorescent and dark pigment; Supplementary Table S1). In addition to antioxidant enzymes, the synthesis of pigments with radical scavenging properties represents an alternative metabolic response able to promote bacterial survival and persistence (Orlandi et al., 2015; Thippakorn et al., 2018).

In accordance with these results, proteins associated with pyomelanin synthesis from homogentisate (HGA) were revealed, and, most of them were inhibited by the treatment (Supplementary Tables S3, S4 and Figure 2). To date, and to the best of our knowledge, pyomelanin related genes and proteins were previously reported only for *P. aeruginosa* (Hunter and Newman, 2010); production of pyomelanin in *P. aeruginosa* contributes to increased resistance to oxidative stress and persistence in chronic infections (Rodríguez-Rojas et al., 2009; Hocquet et al., 2016). The inhibitory effect of HLF on HGA synthesis, putatively explains the absence of the gray pigment in the treated culture (Figure 2 and Supplementary Figure S5C). Given the structural similarity between toluene and HGA, MlaD (PROKKA_01066) was associated to the transport of HGA across the cytoplasmic membrane; out of the cell and under aerobic condition, HGA is rapidly oxidized and polymerized into pyomelanin (Hunter and Newman, 2010). Thus, although other studies should be performed, the HLF-induced repression of PROKKA_01520, converting tyrosine in HGA, would suggest the consequent reduction in the transport of this metabolite across the outer the cell and the absence of pyomelanin-like pigment in the supernatant. HGA can be also converted in TCA cycle intermediates by a fumarylacetoacetase (Rodríguez-Rojas et al., 2009); this enzyme (PROKKA_01666) was indeed detected in ITEM 17298 and negatively affected by HLF (Figure 2). Previous experimental trials blocking this latter reaction, but with a consequent pyomelanin hyperproduction, promoted adaptive mutations, such as hyperproduction of alginate, reduced growth rate, increased biofilm formation capacity, reduced expression of exoenzymes and increased antibiotic resistance, suggesting that the production of this pigment contributes to the adaptive advantage of *P. aeruginosa* in chronic infections (Rodríguez-Rojas et al., 2009).

The metabolic route correlated to pyomelanin synthesis crossed the shikimate pathway, producing chorismate from D-erythrose 4-phosphate (PROKKA_03075), a pentose phosphate intermediate; then chorismate is necessarily converted in tryptophan. This latter pathway was recently associated with an indigo derivate pigment by Andreani et al. (2015, 2019), even though the authors failed to assign molar mass. As depicted in Figure 2, the biosynthesis of most proteins of this pathway was inhibited by the treatment with BLF derived peptides. In addition to these putative pigments, in our previous studies (Caputo et al., 2015; Quintieri et al., 2019b) we also found the colorless and reduced form of 3’,3’-bipyridyl pigment indigoidine (leucoindigoidine, m/z = 251.0781), and its synthesis-related proteins (PROKKA_02722 and PROKKA_00418; Reverchon et al., 2002; Takahashi et al., 2007; Beer et al., 2014; Owen et al., 2016). Both these proteins were also identified in this study and their amounts did not change significantly in presence of HLF. In other microorganisms, production efficiency of indigoidine relies on the respiratory metabolic state (Wehrs et al., 2018); in particular the TCA cycle intermediate alpha-ketoglutarate supplies L-glutamine, the precursor for indigoidine formation via the amino acids glutamate and glutamine (Figure 2). Although some metabolic pathways involved in glutamate production were repressed by the treatment (Figure 2), no further hypothesis can be suggested by herein reported data.

*Pseudomonas fluorescens* ITEM 17298 also produced fluorescent colonies on King B (Supplementary Table S1), putatively correlated to the synthesis of fluorescent siderophores, well known as pyoverdines (Pvds) (Budzikiewicz, 1996). Siderophores recover iron from the environment, transfer it across the inner membrane and store iron in cytoplasm until its use to carry out critical cell functions; then, it is plausible to assume that Pvds might sustain *P. fluorescens* ITEM 17298 in its adaptation strategies. As in *P. aeruginosa* and in other *Pseudomonas* spp., in ITEM 17298, genes are closely located in different operons: PvdS synthesis firstly requires the L-2,4-diaminobutyrate from aspartate (PROKKA_01890, PROKKA_05531, PROKKA_04061) and D-tyrosine as intermediates; the product, with an unformed chromophore is, then, transported into periplasm for the maturation step (Ringel and Brüser, 2018). This latter step includes the acylase PvdQ and PvdP (PROKKA_00251 and PROKKA_05376) that were herein exclusively detected in the untreated samples. Besides PvdQ and PvdP, proteins associated with pyoverdin signaling cascade (the TonB receptor-like PROKKA_01603 and PROKKA_04286, the ferripyoverdine receptor PROKKA_05370 and the hemin receptor PROKKA_04650) were repressed in treated cells by suggesting a possible impairment of iron assimilation and adaptive mechanism by the strain (Figure 2). Moreover, Pvds are involved in biofilm formation and virulence by *P. aeruginosa* (Park et al., 2014); pyoverdine synthesis inhibitors, indeed, represent a therapeutic approach to mitigate pathogenesis (Kirienko et al., 2018).

### Proteins Involved in Pathogenesis

*Pseudomonas fluorescens* is present in the human gut as a low-level commensal (Caputo et al., 2018); however, it is not generally considered a bacterial pathogen in human. Recently, some Authors suggested the association between this species and some human diseases (Scales et al., 2014; Biaggini et al., 2015; Nishimura et al., 2017), bringing to attention a problem not considered until now. Although in this work ITEM 17298 was grown at low temperature, the occurrence of proteins putatively involved in pathogenesis were identified. These proteic determinants could be also involved in the microbial competition and adaptation to environmental stresses. It is worth highlighting that pseudomonads become the dominant population in cold stored foods (Baruzzi et al., 2012, 2015; Carminati et al., 2019). In addition, the switch from planktonic to biofilm state causes physiological, metabolic, and phenotypic changes that can also favor virulence factor production and infection strategies, as widely demonstrated for *P. aeruginosa* (Hall-Stoodley et al., 2004).

In particular, in ITEM 17298 the genomic locus comprising PROKKA_03297-03312 is homolog to the gene cluster encoding the type VI secretion system T6SS, of which the core components consisted of 13 genes conserved in *P. aeruginosa* (80% of nucleotide similarity), *V. cholerae*, and *Acinetobacter* (Chen et al., 2015). In *P. aeruginosa*, this secretion system was structurally homolog to the puncturing device of bacteriophage tail: it is responsible for delivering effector proteins (peptidases, phospholipase D, toxins) in host cells or exhibits antibacterial activity against other bacteria to survive and to prevail in the environmental reservoir (Chen et al., 2015). Under HLF treatment, some T6SS constituting proteins were repressed (Figure 2 and Supplementary Table S3). Among these, HLF repressed PROKKA_03301 (hemolysin co-regulated protein), playing an important role in competition with other bacteria, as demonstrated in environmental *P. fluorescens* (Decoin et al., 2015); however, the implication in other mechanisms by *P. fluorescens* should not be excluded.

Except for *Pseudomonas* spp. of agriculture interest, no studies have been reported for investigating virulence of pseudomonads spoilers. Likewise *P. aeruginosa*, also *P. fluorescens* has a variety of genes for the production of siderophores (Supplementary Table S5), as well as T1SS and T6SS responsible for introducing the virulence proteins into the cytoplasm of eukaryotic cells. The difference in the degree of virulence and specificity of both bacteria suggests the presence of a wide range of effector proteins correlated with these secretion systems (Coombes, 2009). A putative effector of T1SS was PROKKA_00834, the Alkaline protease AprA, a virulence factor secreted by *P. aeruginosa* known to cause serious infection in patients with cystic fibrosis, cancer and severe burns (van‘t Wout et al., 2015); this enzyme was detected in untreated ITEM 17298, whilst it was absent in HLF treated cells (Table 1).

In addition to AprA, also the effector hemolysin precursor (SlhA, PROKKA_05595), belonging to filamentous hemagglutinin family, was repressed by HLF treatment. SlhA was previously found to elicit eukaryotic cells lysis and the autophagic response by *S. marcescens* in host cells (Di Venanzio et al., 2014), protecting bacteria from degradative enzymes and immune responses, but also providing nutrients from cellular debris. Its role in infected *Galleria* larvae mortality was also recently investigated for other bacteria (Brothers et al., 2019), and it is a known virulence determinant in several pathogenesis models. No information can be provided regarding the role of SlhA on increased *Galleria* mortality by ITEM 17298; however, the secretion system type V ShlB, required for the secretion of ShlA hemolysin across the outer membrane, was also found in ITEM 17298 (PROKKA_04561) grown at higher temperature (30°C; Quintieri et al., 2019b). In addition to this, ShlA have been defined as important virulence factor that modulates pathogenesis by this species in aquaculture animals (Sun et al., 2016).

## Conclusion

In this work for the first time we showed that distinctive traits (biofilm and pigments) of psychrotrophic *Pseudomonas* spp. food spoilers could be involved in adaptive responses to cold stresses and in microbial competition. In fact, comparative proteomic analyses on *P. fluorescens* ITEM 17298, treated and untreated with antibiofilm BLFPs, revealed several pathways involved in its spoilage behavior and pathogenesis. This latter was also confirmed by the survival reduction of infected *G. mellonella* larvae. The results of this work also pave the way to the development of novel strategies to control the spread of psychrotrophic food spoilage pseudomonads. To this aim, bovine lactoferrin derived peptides confirm its efficacy as antibiofilm agents to be applied for further food and clinical studies.

## Data Availability Statement

The raw data supporting the conclusions of this article will be made available by the authors, without undue reservation, to any qualified researcher.

## Author Contributions

LQ and KR designed the research. FF performed the genomic analysis. LQ and DZ performed the proteomic analysis and analyzed the proteomic data. DA performed the mass spectrometry analyses. LQ and LC performed and analyzed the microbiological data. LC performed the pathogenicity test. LQ, LC, and FF wrote the manuscript. LQ, AL, LC, FF, and DZ revised the manuscript.

## Conflict of Interest

The authors declare that the research was conducted in the absence of any commercial or financial relationships that could be construed as a potential conflict of interest.

## Footnotes

1 http://blast.ncbi.nlm.nih.gov/Blast.cgi

2 www.prophane.de

3 http://www.decodon.com/

## Supplementary Material

FIGURE S1 Biofilm biomass produced by *Pseudomonas* spp., grown at 15°C in M63 after 24, 48, and 72 h. Values were determined by measuring the absorbance of Crystal Violet at 570 nm (O’Toole, 2011). Error bars represent the standard error measured among three independent replicates. Values with different superscript letters are significantly different (*P* < 0.05; *post hoc* Tukey HSD test).

FIGURE S2 Colony morphologies of *P. fluorescens* ITEM 17298, ITEM 17299 and NCPPB 1964 on Congo Red agar plates (panel **A**), and M63 agar plates, containing 0.3% of agar and Congo Red dye (swimming assay), after 72 h of incubation at 15°C (panel **B**).

FIGURE S3 Motility assays performed at 15°C for 24, 48, and 72 h. Swarming **(A)** and swimming motility **(B)** of 4 *Pseudomonas* spp. biofilm producers (ITEM 17298, ITEM 17299, PS36, and NCPPB 1964) and two negative biofilm producers (PS37 and PS4) in M63 medium. Values represent the mean diameter of corresponding motility zones. Twitching motility **(C)** of *P. fluorescens* ITEM 17298 in M63 medium. Values were determined by measuring the absorbance of Crystal violet (CV) at 570 nm. Bars represent the average ± the standard deviation (*N* = 3). Ranked significantly different (*P* < 0.05) means were separated according to a Kruskal–Wallis test [**(A)** *H* = 47.637, df = 17, *P* < 0.0001; **(B)** *H* = 50.692, df = 17, *P* < 0.0001; **(C)** *H* = 52.704, df = 17, *P* < 0.0001] followed by Games and Howell’s multiple comparisons test in order to assign different letters.

FIGURE S4 Swarming motility of 4 *Pseudomonas* spp. biofilm producers (ITEM 17298, ITEM 17299, PS36, and NCPPB 1964) and two negative biofilm producers (PS37 and PS4) in M63 medium after incubation for 24 and 72 h at 15°C.

FIGURE S5 **(A)** Bacterial counts (expressed as Log CFU/mL) of *P. fluorescens* ITEM 17298 in M63 broth in the absence (UT) or presence (T) of HLF (0.75, 1.5, or 3 mg/mL) at 15°C for 72 h of incubation. Values represent the average ± the standard deviation (*n* = 3). No statistically significant differences (*P* > 0.05) were found among values at each incubation time for both assayed temperatures according to HSD Tukey’s test. **(B)** Biofilm biomass produced by *P. fluorescens* ITEM 17298, grown in presence of sublethal concentrations of HLF at 15°C for 72 h. Values were determined by measuring the absorbance of Crystal violet at 570 nm (O’Toole, 2011). Error bars represent the standard error measured among three independent replicates; **(C)** discoloration of liquid cultures in M63 of untreated or HLF-treated ITEM 17298 (0.75, 1.5 or 3 mg/mL) after 72 h of incubation at 15°C.

FIGURE S6 Genomic organization of alginate operon in *P. aeruginosa* and *P. fluorescens* ITEM 17298. Gene clustering is represented by the arrows superposed on the black horizontal line. Intergenic spaces are not drawn in scale; *argF*, ornithine carbamoyltransferase; PA3538, ABC transporter ATP-binding protein; yaaA, peroxide stress protein; *algD*, GDP-mannose 6-dehydrogenase AlgD; *alg8*, glycosyltransferase Alg8; *alg44*, alginate biosynthesis protein Alg44; *algK*, alginate biosynthesis protein AlgK; *algE*, alginate production protein AlgE; *algG*, alginate-c5-mannuronan-epimerase; *algX*, alginate biosynthesis protein AlgX; *algL*, alginate lyase precursor; *algI*, alginate O-acetyltransferase AlgI; *algJ*, alginate O-acetyltransferase AlgJ; *algF*, alginate O-acetyltransferase AlgF; *algA*, Alginate biosynthesis protein AlgA; *arnB*, UDP-4-amino-4-deoxy-L- arabinose–oxoglutarate aminotransferase; *arnC*, undecaprenyl-phosphate 4-deoxy-4-formamido-L-arabinose transferase; *arnA*, bifunctional UDP-glucuronic acid decarboxylase/UDP-4-amino-4-deoxy-L-arabinose formyltransferase; *arnD*, 4-deoxy-4-formamido-L-arabinose-phosphoundecaprenol deformylase ArnD; *arnT*, 4-amino-4-deoxy-L-arabinose lipid A transferase; *arnE*, 4-amino-4-deoxy-L-arabinose-phosphoundecaprenol flippase subunit ArnE; *arnF*, 4-amino-4-deoxy-L-arabinose-phosphoundecaprenol flippase subunit *ArnF*; PA3559, nucleotide sugar dehydrogenase; fbpc, Fe(3+) ions import ATP-binding protein FbpC; *pho-H*, PhoH-like protein; *pdaC*, peptidoglycan-N-acetylmuramic acid deacetylase *PdaC*; PROKKA_01713, signal peptide; *rhsC*, deoxyribonuclease RhsC.

TABLE S1 Colony appearance of *Pseudomonas* spp. strains grown on selective media (King A, King B, and M63) for 5 days at 15°C. C, cream; B, brown; F, fluorescent green; D, dark; P, pink.

TABLE S2 EPS production by selected *Pseudomonas* spp. strains: panel **A**) calibration curve by measuring the OD 490 nm of Congo Red (CR) at 40, 20, 10, 5, and 2.5 μg/mL. Panel **(B)** absorbance values of unbound Congo Red registered in each strain culture after incubation at 15°C for 72 h.

TABLE S3 Quantitative proteome data MM, based on NSAF.

TABLE S4 List of proteins involved in pyomelanin pathway.

TABLE S5 List of protein involved in pyoverdine metabolism. ^∗^NA, not annotated.

## Abbreviations

1D-SDS PAGE: one-dimensional sodium dodecyl sulfate polyacrylamide gel electrophoresis
AMPs: antimicrobial peptides
ANOVA: analysis of variance
BLF: bovine lactoferrin
BLFPs: bovine lactoferrin-derived peptides
CFU: colony-forming unit
FDR: protein false discovery rate
GeLC-MS/MS: in-gel tryptic digestion followed by liquid chromatography-tandem mass spectrometry
GO: gene ontology
HLF: bovine lactoferrin hydrolyzate
KEGG: Kyoto Encyclopedia of Genes and Genomes
MBIC: minimum biofilm inhibitory concentration
MIC: minimal inhibitory concentration
NCBI: National Center for Biotechnology Information
NSAF: normalized spectral abundance factor
QS: quorum sensing
SpC/L: spectral counts/protein length
TE: Tris–Ethylenediaminetetraacetic acid.
