# Supplementary figures and images for: Biofilm and Pathogenesis-Related Proteins in the Foodborne P. fluorescens ITEM 17298 With Distinctive Phenotypes During Cold Storage
Source: Front Microbiol. 2020 May 28;11:991. doi: 10.3389/fmicb.2020.00991 (PMC7326052; doi:10.3389/fmicb.2020.00991)

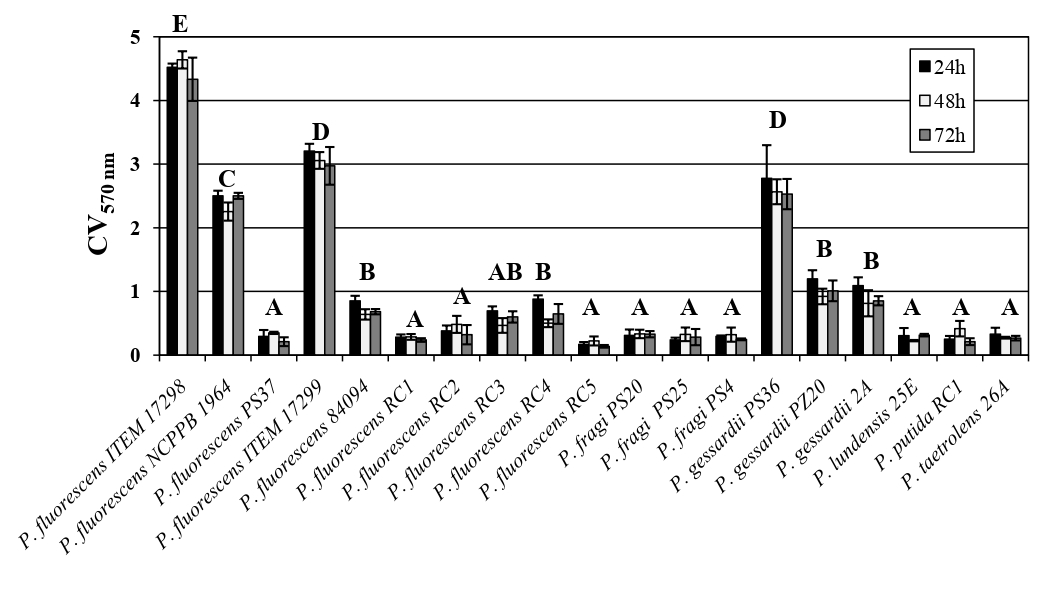

Supplement: FIGURE S1 — Biofilm biomass produced by Pseudomonas spp., grown at 15°C in M63 after 24, 48, and 72 h. Values were determined by measuring the absorbance of Crystal Violet at 570 nm (O’Toole, 2011). Error bars represent the standard error measured among three independent replicates. Values with different superscript letters are significantly different (P < 0.05; post hoc Tukey HSD test). [file Data_Sheet_4.zip › figure s1-s6/Supplementary Figure S1.jpg]

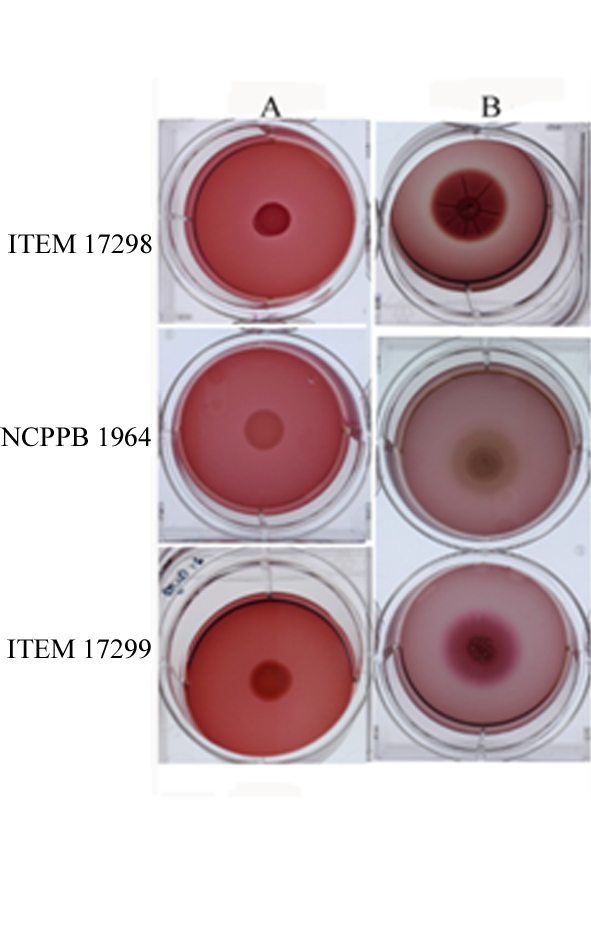

Supplement: FIGURE S1 — Biofilm biomass produced by Pseudomonas spp., grown at 15°C in M63 after 24, 48, and 72 h. Values were determined by measuring the absorbance of Crystal Violet at 570 nm (O’Toole, 2011). Error bars represent the standard error measured among three independent replicates. Values with different superscript letters are significantly different (P < 0.05; post hoc Tukey HSD test). [file Data_Sheet_4.zip › figure s1-s6/Supplementary Figure S2.jpg]

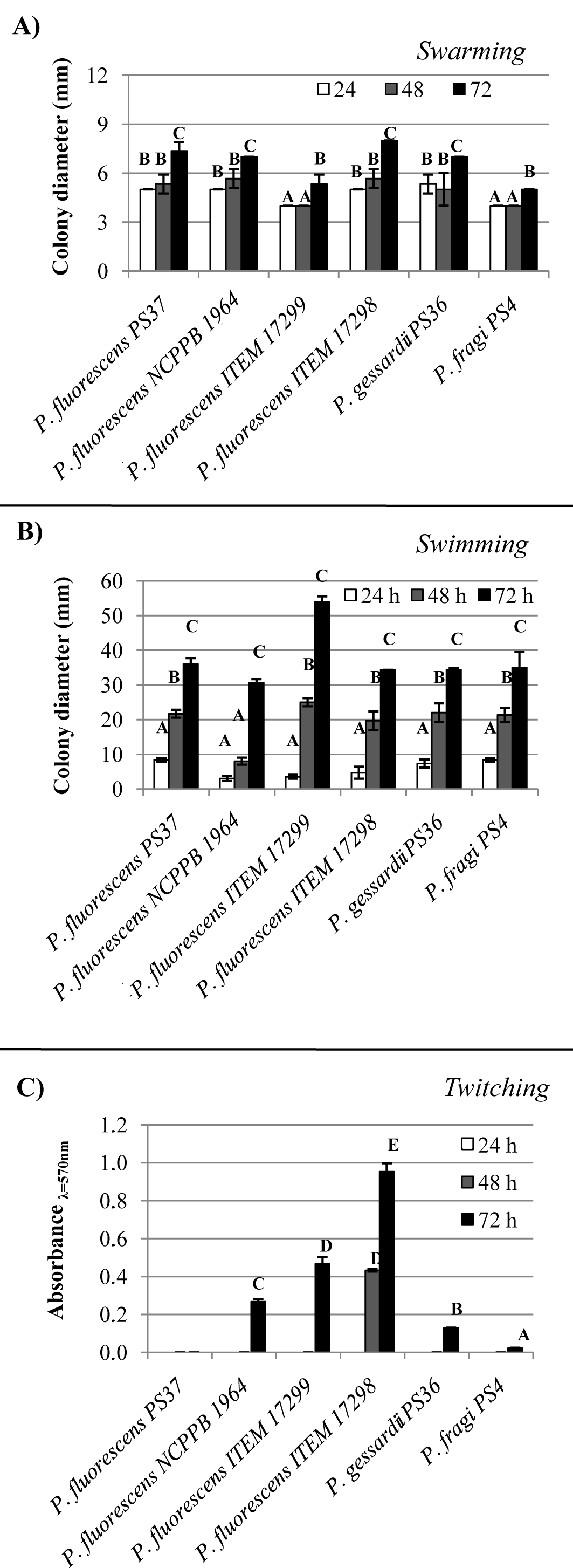

Supplement: FIGURE S1 — Biofilm biomass produced by Pseudomonas spp., grown at 15°C in M63 after 24, 48, and 72 h. Values were determined by measuring the absorbance of Crystal Violet at 570 nm (O’Toole, 2011). Error bars represent the standard error measured among three independent replicates. Values with different superscript letters are significantly different (P < 0.05; post hoc Tukey HSD test). [file Data_Sheet_4.zip › figure s1-s6/Supplementary Figure S3.tif]

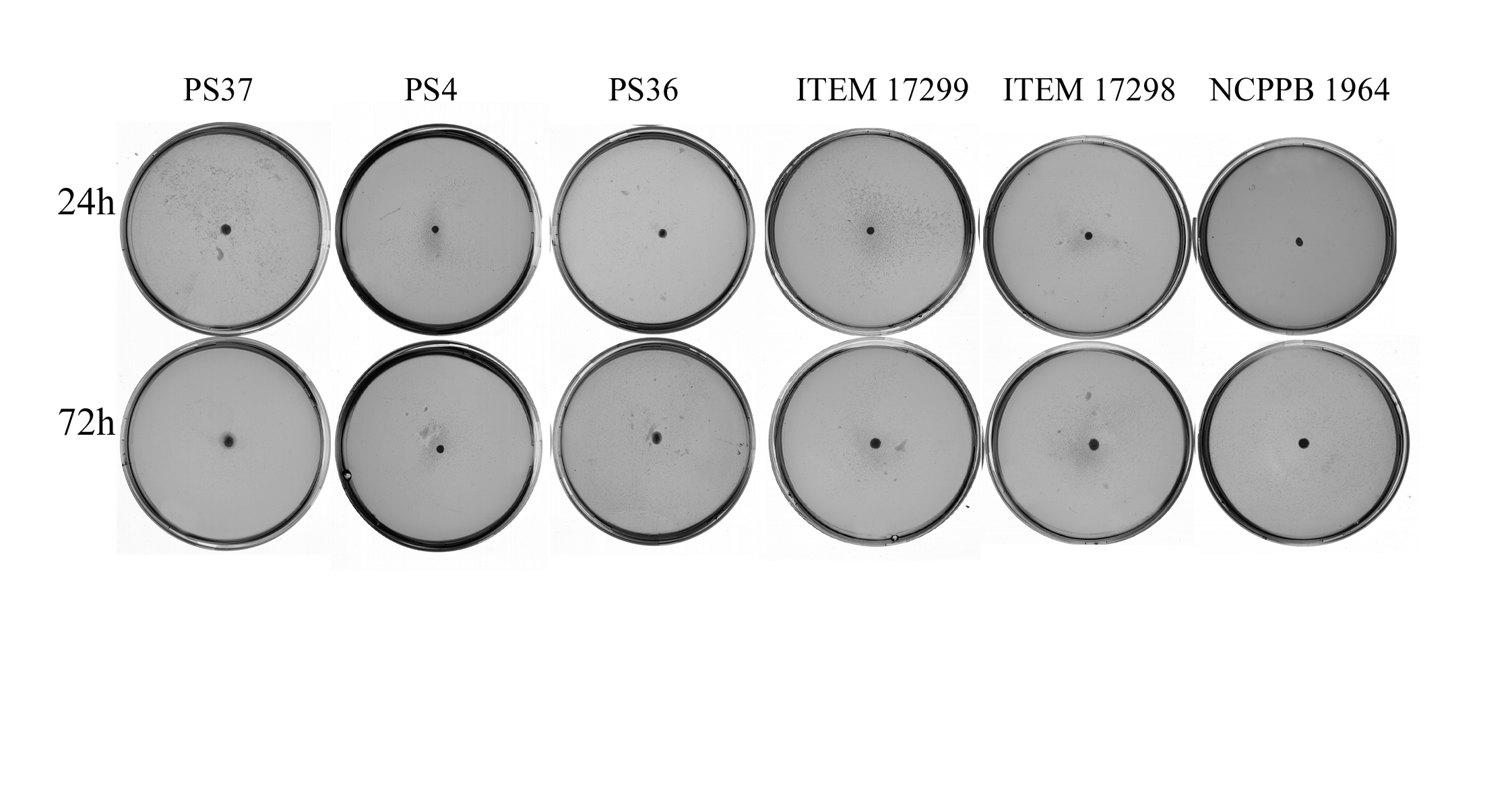

Supplement: FIGURE S1 — Biofilm biomass produced by Pseudomonas spp., grown at 15°C in M63 after 24, 48, and 72 h. Values were determined by measuring the absorbance of Crystal Violet at 570 nm (O’Toole, 2011). Error bars represent the standard error measured among three independent replicates. Values with different superscript letters are significantly different (P < 0.05; post hoc Tukey HSD test). [file Data_Sheet_4.zip › figure s1-s6/Supplementary Figure S4.tif]

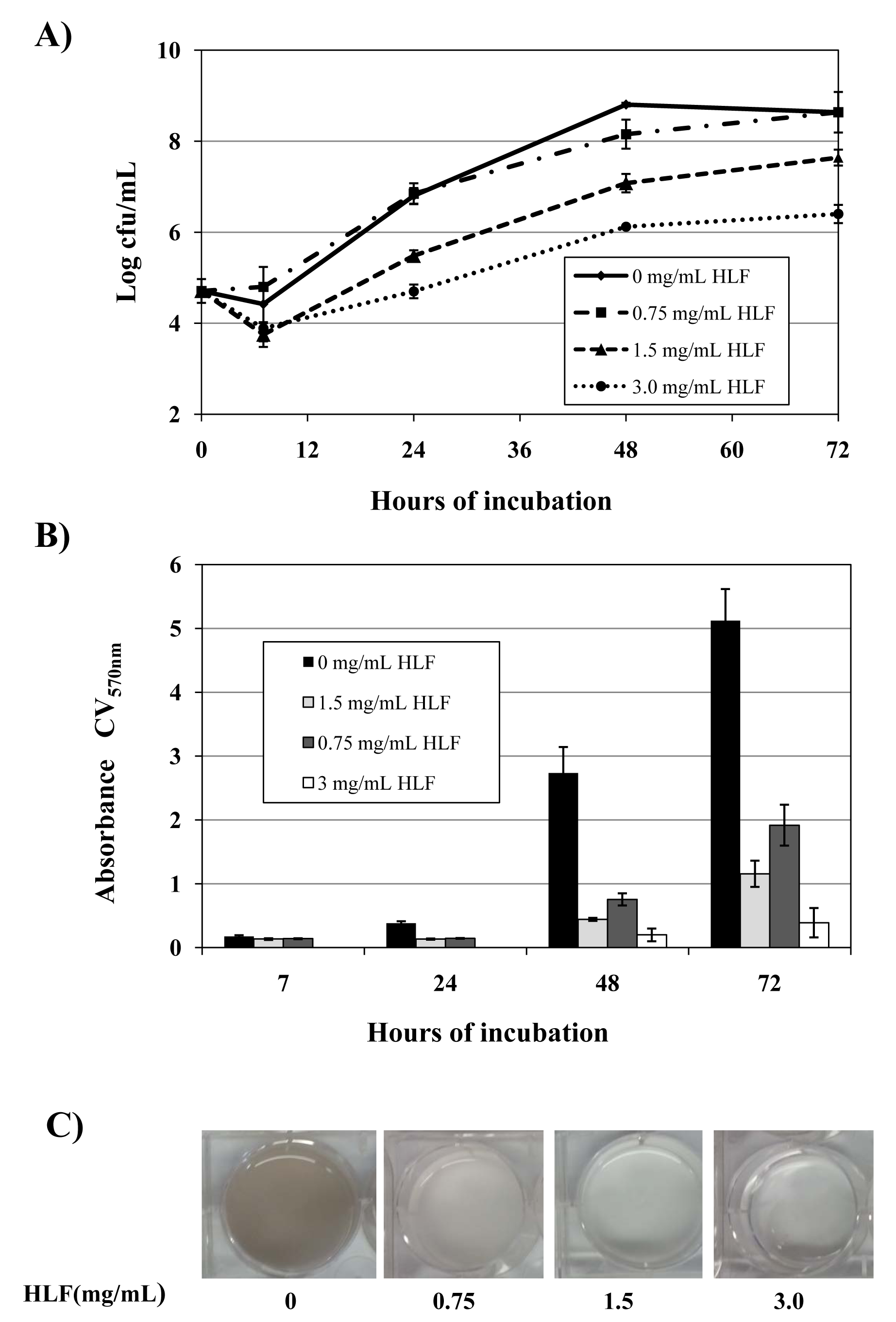

Supplement: FIGURE S1 — Biofilm biomass produced by Pseudomonas spp., grown at 15°C in M63 after 24, 48, and 72 h. Values were determined by measuring the absorbance of Crystal Violet at 570 nm (O’Toole, 2011). Error bars represent the standard error measured among three independent replicates. Values with different superscript letters are significantly different (P < 0.05; post hoc Tukey HSD test). [file Data_Sheet_4.zip › figure s1-s6/Supplementary Figure S5.tif]

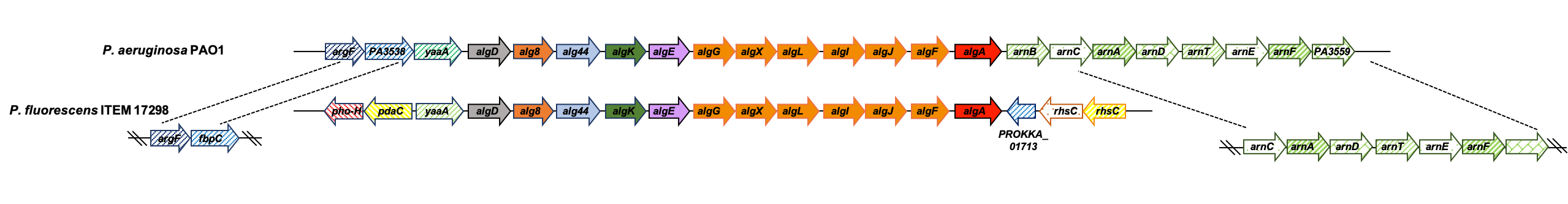

Supplement: FIGURE S1 — Biofilm biomass produced by Pseudomonas spp., grown at 15°C in M63 after 24, 48, and 72 h. Values were determined by measuring the absorbance of Crystal Violet at 570 nm (O’Toole, 2011). Error bars represent the standard error measured among three independent replicates. Values with different superscript letters are significantly different (P < 0.05; post hoc Tukey HSD test). [file Data_Sheet_4.zip › figure s1-s6/Supplementary Figure S6.tif]
