# Supplementary material for: Biofilm and Pathogenesis-Related Proteins in the Foodborne P. fluorescens ITEM 17298 With Distinctive Phenotypes During Cold Storage
Source: Front Microbiol. 2020 May 28;11:991. doi: 10.3389/fmicb.2020.00991 (PMC7326052; doi:10.3389/fmicb.2020.00991)
Supplement: TABLE S1 — Colony appearance of Pseudomonas spp. strains grown on selective media (King A, King B, and M63) for 5 days at 15°C. C, cream; B, brown; F, fluorescent green; D, dark; P, pink. [file Data_Sheet_1.pdf]

Supplementary Tables

Table S1. Colony appearance of *Pseudomonas* spp. strains grown on selective media (King A, King B, and M63) for 5 days at 15°C.

**Species**	**Strains**	**KING A**	**KING B**	**M63**
P. fluorescens	PS37	C	C	C
	84094	**B**	**B**	C
	ITEM 17299	**B**	**B**	**D**
	NCPPB 1964T	C	**F**	C
	ITEM 17298	C	**F**	**D**
	RC1	C	C	C
	RC2	C	C	C
	RC3	C	**F**	C
	RC4	C	**F**	C
	RC5	C	C	C
P. gessardii	PZ20	C	**F**	C
	PS36	**P**	**F**	C
	2A	**P**	**F**	**B**
P. fragi	PS25	C	C	C
	PS4	C	C	C
	PS20	C	C	C
P. taetrolens	26A	C	C	C
P. lundensis	25E	C	C	C
P. putida	RC10	C	C	C

C, cream; B, brown; F, fluorescent green; D, dark; P, pink.

Table S4. List of proteins involved in pyomelanin pathway

Gene Name	Protein ID	Product
aroE	PROKKA_00590	shikimate 5-dehydrogenase
trpE	PROKKA_04707	anthranilate synthase component I
trpB	PROKKA_00578	tryptophan synthase subunit beta
trpC	PROKKA_04711	indole-3-glycerol-phosphate synthase
phhA	PROKKA_01785	phenylalanine 4-monooxygenase
phhB	PROKKA_01786	pterin-4-alpha-carbinolamine dehydratase
phhC	PROKKA_01787	aromatic-amino-acid aminotransferase
pyrE	PROKKA_03334	orotate phosphoribosyltransferase
pyrC	PROKKA_04036	dihydroorotase
pyrD	PROKKA_02015	dihydroorotate dehydrogenase 2
lolA	PROKKA_02658	outer membrane lipoprotein carrier protein LolA
ispH	PROKKA_03878	4-hydroxy-3-methylbut-2-enyl diphosphate reductase
tyrB	PROKKA_01326	aromatic-amino-acid aminotransferase
hpd	PROKKA_05182	4-hydroxyphenylpyruvate dioxygenase
hmgA	PROKKA_01667	homogentisate 1,2-dioxygenase
fahA	PROKKA_01666	fumaryl acetoacetase
zwf	PROKKA_05652	glucose-6-phosphate dehydrogenase
aroB	PROKKA_03075	NAD+ dependent 3-dehydroquinate synthase
hatA/mlaF	PROKKA_01064	ABC transporter ATP-binding protein
hatB/mlaE	PROKKA_01065	phospholipid ABC transporter permease protein MlaE
hatC/mlaD	PROKKA_01066	phospholipid ABC transporter-binding protein MlaD
hatD/mlaC	PROKKA_01067	phospholipid-binding protein MlaC precursor
hatE	PROKKA_01068	hypothetical protein

Table S5. List of protein involved in pyoverdine metabolism

Gene Name	ITEM 17298 Protein ID	Product	Ref seq *P. aeruginosa* PAO-1	Protein Identity (%)
pvdQ	PROKKA_00251	3-oxo-C12-homoserine lactone acylase PvdQ	NP_251075.1	55
ccmF	PROKKA_00362	cytochrome C-type biogenesis protein CcmF	NP_250171.1	85
ccmC	PROKKA_00365	heme exporter protein CcmC	NP_250168.1	86
cc4	PROKKA_00533	cytochrome c4 precursor	NP_254177.1	79
pvdS	PROKKA_01590	Sigma factor PvdS, controling pyoverdin biosynthesis	NP_251116.1	90
pvdL	PROKKA_01592	peptide synthase	NP_251114.1	74
opmQ	PROKKA_02491	probable outer membrane protein precursor	NP_251081.1	62
pvdT	PROKKA_02492	Pyoverdin biosynthesis protein PvdT	NP_251080.1	81
pvdR	PROKKA_02493	Pyoverdin biosynthesis protein PvdR	NP_251079.1	70
fpvl	PROKKA_02494	Sigma-70 factor FpvI	NP_251077.1	75
pvdA	PROKKA_02495	L-ornithine 5-monooxygenase	NP_251076.1	75
	PROKKA_04133	conserved hypothetical protein	NP_251102.1	85
pvdH	PROKKA_04134	L-2,4-diaminobutyrate:2-ketoglutarate 4-aminotransferase, PvdH	NP_251103.1	83
fpvF	PROKKA_04601	ABC transporter in pyoverdin gene cluster, periplasmic component	NP_251100.1	57
fpvE	PROKKA_04602	ABC transporter in pyoverdin gene cluster, permease component	NP_251099.1	89
fpvD	PROKKA_04603	ABC transporter in pyoverdin gene cluster, ATP-binding component	NP_251098.1	75

fpvK	PROKKA_04605	Hypothetical protein in pyoverdin gene cluster	NP_251096.1	49
fpvJ	PROKKA_04606	Hypothetical protein in pyoverdin gene cluster	NP_251095.1	78
fpvG	PROKKA_04608	Hypothetical protein FpvG	NP_251093.1	70
pcpS	PROKKA_05097	4'-phosphopantetheinyl transferase	NP_249856.1	65
fpvA	PROKKA_05370	ferripyoverdine receptor	NP_251088.1	66
pvdE	PROKKA_05371	pyoverdine biosynthesis protein PvdE	NP_251087.1	81
pvdF	PROKKA_05372	pyoverdine synthetase F	NP_251086.1	79
pvdO	PROKKA_05373	pyoverdine biosynthesis protein PvdO	NP_251085.1	73
pvdN	PROKKA_05374	Pyoverdin biosynthesis protein PvdN, putative aminotransferase, class V	NP_251084.1	61
pvdM	PROKKA_05375	Putative dipeptidase, pyoverdin biosynthesis PvdM	NP_251083.1	76
pvdP	PROKKA_05376	pyoverdine biosynthesis protein pvdP	NP_251082.1	67
lysC	PROKKA_01890	Aspartokinase	NP_249595.1	89
asd	PROKKA_05531	Aspartate-semialdehyde dehydrogenase	NP_251807.1	90
doeD	PROKKA_04061	Diaminobutyrate-2-oxoglutarate aminotransferase	NA*	-
tonB	PROKKA_01603	putative TonB-dependent receptor precursor	NP_249472.1	71
tonB	PROKKA_04286	putative TonB-dependent receptor BfrD precursor	NP_249125.1	63
phuR	PROKKA_04650	Hemin receptor precursor	NP_253398.1	65

*NA: *not annotated*
